# Validation of an Ion Torrent Sequencing Platform for the Detection of Gene Mutations in Biopsy Specimens from Patients with Non-Small-Cell Lung Cancer

**DOI:** 10.1371/journal.pone.0130219

**Published:** 2015-06-15

**Authors:** Shiro Fujita, Katsuhiro Masago, Jumpei Takeshita, Chiyuki Okuda, Kyoko Otsuka, Akito Hata, Reiko Kaji, Nobuyuki Katakami, Yukio Hirata

**Affiliations:** Division of Integrated Oncology, Institute of Biomedical Research and Innovation, Chuo-ku, Kobe, 650–0047, Japan; Queen Mary Hospital, HONG KONG

## Abstract

**Background:**

Treatment for patients with advanced non-small cell lung cancer (NSCLC) is often determined by the presence of biomarkers that predict the response to agents targeting specific molecular pathways. Demands for multiplex analysis of the genes involved in the pathogenesis of NSCLC are increasing.

**Methods:**

We validated the Ion Torrent Personal Genome Machine (PGM) system using the Ion AmpliSeq Cancer Hotspot Panel and compared the results with those obtained using the gold standard methods, conventional PCR and Sanger sequencing. The cycleave PCR method was used to verify the results.

**Results and Conclusion:**

The Ion Torrent PGM resulted in a similar level of accuracy in identifying multiple genetic mutations in parallel, compared with conventional PCR and Sanger sequencing; however, the Ion Torrent PGM was superior to the other sequencing methods in terms of increased ease of use, even when taking into account the small amount of DNA that was obtained from formalin-fixed paraffin embedded (FFPE) biopsy specimens.

## Introduction

Treatment for patients with advanced non-small cell lung cancer (NSCLC) is often determined, as is the case for other human cancers, by the presence of biomarkers that predict the response to agents targeting specific molecular pathways in malignant cells. Favorable response to tyrosine kinase (TK) inhibitors targeting the epidermal growth factor receptor (EGFR), in cases of NSCLC in which mutations are present in the EGFR gene, illustrate the importance of the identification of molecular biomarkers, and several such oncogenic alterations has been reported to date [1–3]. These molecular alterations can be divided into two categories; genetic rearrangements that require RNA-based analysis and/or fluorescence in situ hybridization for their identification, and gene mutations (including small insertion or deletion events) that are investigated using DNA-based analysis. Mutations have been discovered in several genes in addition to *EGFR* that are involved in the pathogenesis of NSCLC (i.e. *KRAS*, *NRAS*, *HER2*, *AKT1*, *BRAF*, *PIC3CA*) and the demand for multiplex analysis of these genes is increasing.

The only tissue specimens that are usually available for mutational analysis in the clinical setting are biopsy specimens obtained transbronchially or transcutaneously. These samples tend to be small and are subjected to the formalin-fixation process. Detection of mutations by performing PCR and Sanger sequencing in parallel is time consuming and requires a substantial amount of DNA, which is often unavailable due to the small sample size. A recently developed technique, massively parallel DNA sequencing, allows fast, sensitive and highly specific detection of gene mutations in a single assay, at reasonable cost.

Here, we used the Ion Torrent Personal Genome Machine (PGM) system (Thermo Fisher Scientific) in conjunction with the Ion AmpliSeq Cancer Hotspot Panel, version 2 and compared the results with those obtained by the gold standard methods for mutational analysis, conventional PCR and Sanger sequencing. Furthermore, a highly sensitive PCR method, the cycleave PCR method, was employed, focusing on EGFR and KRAS gene mutations specifically, to verify the results obtained by both conventional PCR and the Ion Torrent PGM system.

## Methods

### Ethics

This study was approved by the Institute of Biomedical Research and Innovation Hospital’s Institutional Review Board. All patients provided written, informed consent. The study was conducted in accordance with the ethical principles of the Declaration of Helsinki.

### Patient information

Tumor samples used in the study were collected from the Institute of Biomedical Research and Innovation Hospital, Japan. In accordance with the current guidelines, all FFPE tumor samples from non-small-cell lung cancer patients with unknown genotype (*EGFR* and *KRAS*) were analyzed by conventional PCR and Sanger sequencing to determine further treatment. As a comparison, a total of 21 tumor samples was analyzed with the Ion PGM system and the cycleave PCR method.

### Analyzed genes

*EGFR*: Exon 19 deletion (including E746 –A750), Exon 21 L858R, Exon 21 L861Q

*KRAS*: Exon 2 G12X

### Tissue samples and DNA Isolations

**Fig 1:**
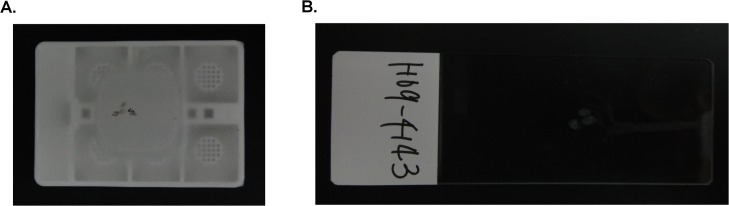
Typical specimen. (A) Formalin-fixed paraffin embedded specimen. (B) One sample of 5-μm-thick section.

Only those biopsy specimens which revealed NSCLC pathologically were analyzed. In all cases, bronchoscopic biopsy or core needle biopsy was performed (Fig 1A and 1B). A dedicated 21-gauge needle was used to obtain histologic core. Histologic cores were fixed with formalin and used for pathologic diagnosis. After histopathologic diagnosis, FFPE samples (1 to 3 samples of 5-μm-thick section) were deparaffinized using xylene. DNA was isolated from the sections using the QIAamp DNA Mini Kit (Qiagen), as per the manufacturer’s instructions. We measured DNA concentration with NanoDrop Lite Spectrophotometer (Thermo Fisher Scientific).

### Conventional PCR and Sanger sequencing

Exons 18 to 21 were amplified by PCR and analyzed. Primers and cycling conditions for PCR amplification were modified from methods published previously [4]. Sequencing reactions were electrophoresed on an ABI PRISM 3100 (Thermo Fisher Scientific). Direct sequencing of the PCR products was carried out in both sense and antisense directions.

### The cycleave PCR method

We used a chimeric DNA-RNA-DNA probe labeled with a fluorescent dye and quencher at each end [5]. A RNA sequence of the probes corresponds to that of the wild type and point mutation (Exon 21 L858R, Exon 21 L861Q) labeled with FMA and ROX, respectively. When mutant molecules are present in the sample and PCR-amplified DNA generates a complete hybrid with the RNA portion of the mutant probe, RNase-H digests the probe at the RNA-DNA heteroduplex into two pieces, leading to a significant increase in fluorescence intensity by separation of the fluorescent dye from the quencher.

To detect the deletion in exon 19 of the EGFR gene, common fragment analysis was used. Sample DNA was amplified with an FAM-labeled primer set and PCR products were electrophoresed. PCR amplified the shorter segment of DNA, creating a new peak in an electropherogram when a deletion mutation was present [5].

### Ion Torrent PGM Library Preparation and Sequencing

An Ion Torrent adapter-ligated library was generated following the manufacturer’s Ion AmpliSeq Library Kit 2.0 protocol (Thermo Fisher Scientific, Rev. 5; MAN0006735). Briefly, 50-ng pooled amplicons were end-repaired, and Ion Torrent adapters P1 and A were ligated with DNA ligase. Following AMPure bead purification (Beckman Coulter), the concentration and size of the library were determined using the Life Technologies StepOne system (Thermo Fisher Scientific) and Ion Library TaqMan Quantitation Assay Kit (Thermo Fisher Scientific).

Sample emulsion PCR, emulsion breaking, and enrichment were performed using the Ion PGM IC 200 Kit (Thermo Fisher Scientific), according to the manufacturer’s instructions. Briefly, an input concentration of one DNA template copy/Ion Sphere Particles (ISPs) was added to the emulsion PCR master mix and the emulsion was generated using the Ion Chef (Thermo Fisher Scientific). Template-positive ISPs were enriched and sequencing was undertaken using 314 BC chips on the Ion Torrent PGM for 65 cycles and barcoding was performed using the Ion DNA Barcoding kit (Thermo Fisher Scientific).

### Variant calling

Data from the PGM runs were initially processed using the Ion Torrent platform-specific pipeline software, Torrent Suite, to generate sequence reads, trim adapter sequences, filter, and remove poor signal-profile reads. Initial variant calling from the Ion AmpliSeq sequencing data was generated using Torrent Suite Software v4.0 with a plug-in ‘‘variant caller” program. To eliminate erroneous base calling, three filtering steps were used to generate final variant calling. The first filter was set at an average depth of total coverage of >100, an each variant coverage of >20, and P-value <0.01. The second filter was employed by visually examining mutations using Integrative Genomics Viewer software (http://www.broadinstitute.org/igv) or CLC Genomics Workbench version 7.5.1 (Qiagen), as well as by filtering out possible strand-specific errors; i.e., a mutation was detected only in either the ‘‘plus” or ‘‘minus” strand, but not in both strands of DNA.

## Results and Discussion

**Fig 2:**
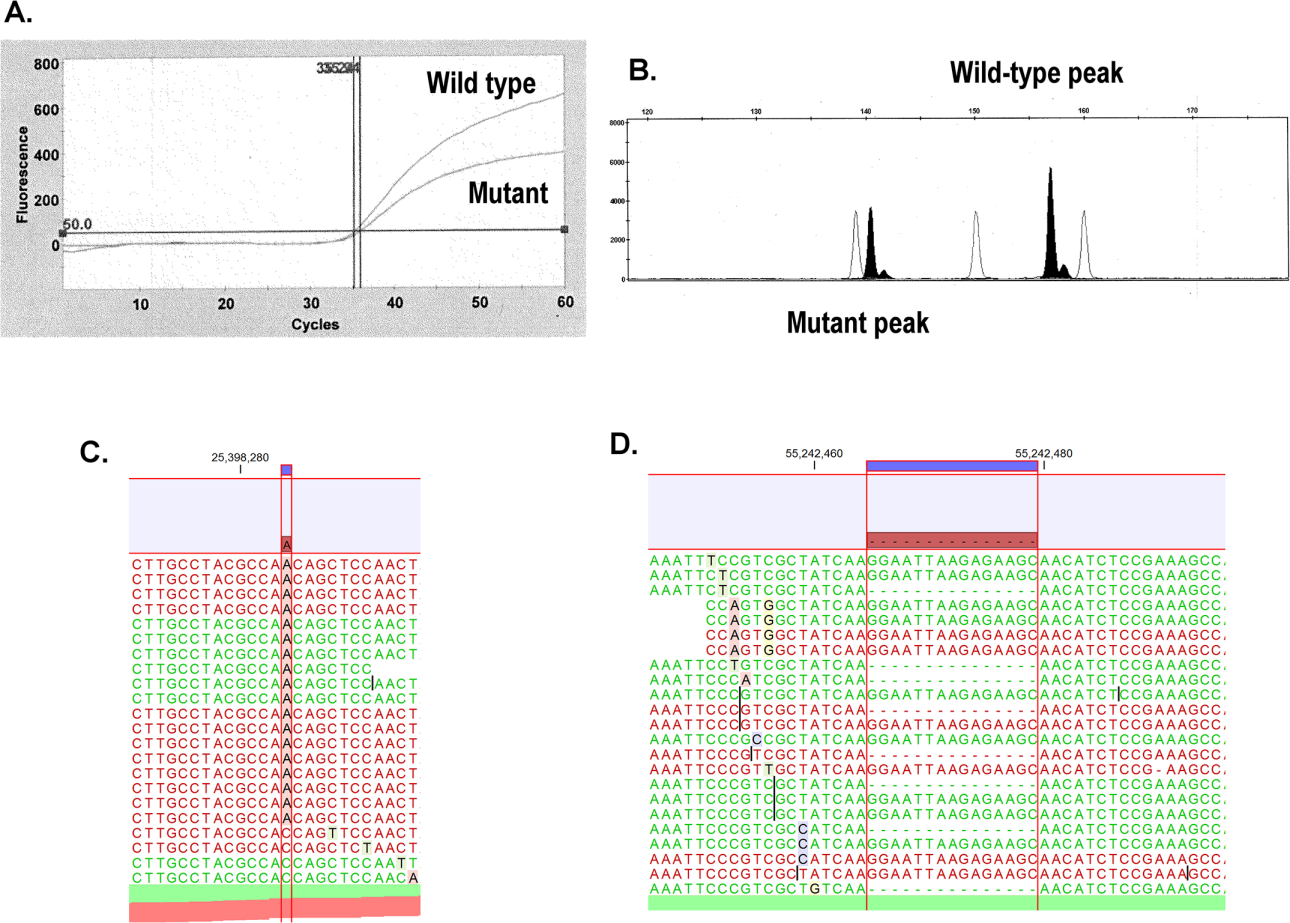
Typical results of analysis. (A) KRAS mutation (G12V) identified by cycleave technology. (B) EGFR mutation (exon 19 deletion) identified by the fragment analysis (C) KRAS mutation (G12V) was detected with Ion PGM technology. C to A transversion was identified. (D) EGFR mutation (exon 19 deletion) was detected with Ion PGM technology.

A total of 21 tumor samples (10 from bronchoscopic transbronchial biopsy, 6 CT-guided tumor biopsy and 5 core-needle superficial lymph node biopsy) were analyzed. A median value of extracted DNA concentration was 115.2 ng/μl (minimum 29.3, maximum 786.1) and an appropriate amount of DNA was used for analysis. As shown in Table 1 and Fig 2, the results of gene mutation analyses obtained by Sanger sequencing were identical in all cases to those derived from analyses using the Ion PGM system. With regard to EGFR, analytical results obtained by Sanger sequencing completely matched those obtained by the cycleave method and the Ion PGM system. None of the tumors examined had mutations in both the EGFR TK domain and the KRAS gene.

**Table 1:** *EGFR* and *KRAS* mutation status.

Gene	Mutation	Number
*EGFR*	Wild	9
	Exon 19 deletion	6
	E746-A750del	5
	E746-T751delinsA	1
	Exon 20 T790M	2
	Exon 21 L858R	5
	Exon 21 L861Q	1
*KRAS*	Wild	16
	G12C	1
	G12D	1
	G12V	3

A second EGFR mutation in which methionine is substituted for threonine at position 790 (T790M) correlates with acquired resistance to EGFR tyrosine kinase inhibitors [6]. In our series, 3 patients underwent a second biopsy after progression on the treatment of EGFR tyrosine kinase inhibitors. DNA sequence of these biopsy specimens by conventional Sanger sequencing showed T790M mutation in two cases. Sequencing using the Ion PGM revealed T790M mutation also in two cases. In addition, among 900 reads obtained, the T790M mutation was observed in 53 reads (5.9%) in the third patient. Although the detected number of reads was below the threshold level, it is possible that the emergence of T790M is the primary cause of resistance to EGFR tyrosine kinase inhibitor treatment in this case.

Despite the small number of cases analyzed, the results suggest that the Ion Torrent PGM is a practicable and sensitive procedure that would be suitable in clinical practice, when only a small amount of malignant tissue is available and multiplex analyses of genes are necessary for treatment planning. In addition to the investigation of gene mutations performed here, further information about genetic rearrangements should to be integrated into current knowledge to improve the treatment of NSCLC and facilitate further study.
